# Supplementary material for: Evaluation of the effects of indocyanine green administration dose and speed on blood concentration and fluorescence in thoracoscopic segmentectomy
Source: PLoS One. 2026 Mar 26;21(3):e0345951. doi: 10.1371/journal.pone.0345951 (PMC13020837; doi:10.1371/journal.pone.0345951)
Supplement: Attachment — Submitted filename: Rebuttal Letter.docx [file pone.0345951.s001.docx]

**PONE-D-25-54320**

**Effects of indocyanine green administration dose and injection speed on blood concentration and fluorescence in thoracoscopic segmentectomy: a two-stage, single-center study**

*PLOS ONE*

Journal Requirements:

When submitting your revision, we need you to address these additional requirements.

1. Please ensure that your manuscript meets PLOS ONE's style requirements, including those for file naming. The PLOS ONE style templates can be found at https://journals.plos.org/plosone/s/file?id=wjVg/PLOSOne_formatting_sample_main_body.pdf and https://journals.plos.org/plosone/s/file?id=ba62/PLOSOne_formatting_sample_title_authors_affiliations.pdf

**Answer: We have confirmed this file naming convention as requested.**

2. PLOS requires an ORCID iD for the corresponding author in Editorial Manager on papers submitted after December 6th, 2016. Please ensure that you have an ORCID iD and that it is validated in Editorial Manager. To do this, go to ‘Update my Information’ (in the upper left-hand corner of the main menu), and click on the Fetch/Validate link next to the ORCID field. This will take you to the ORCID site and allow you to create a new iD or authenticate a pre-existing iD in Editorial Manager.

**Answer: The corresponding author’s ORCID iD is 0000-0003-4098-0579. We will ensure it is validated as required.**

3. When completing the data availability statement of the submission form, you indicated that you will make your data available on acceptance. We strongly recommend all authors decide on a data sharing plan before acceptance, as the process can be lengthy and hold up publication timelines. Please note that, though access restrictions are acceptable now, your entire data will need to be made freely accessible if your manuscript is accepted for publication. This policy applies to all data except where public deposition would breach compliance with the protocol approved by your research ethics board. If you are unable to adhere to our open data policy, please kindly revise your statement to explain your reasoning and we will seek the editor's input on an exemption. Please be assured that, once you have provided your new statement, the assessment of your exemption will not hold up the peer review process.

**Answer: We have uploaded the complete dataset to Figshare and have made it publicly accessible in accordance with the journal’s open data policy. The dataset can be accessed via the following DOI: 10.6084/m9.figshare.30738818.**

4. Thank you for stating the following financial disclosure:

This work was supported by JSPS KAKENHI [Grant Number: JP 22K08976].

Please state what role the funders took in the study. If the funders had no role, please state: "The funders had no role in study design, data collection and analysis, decision to publish, or preparation of the manuscript."

If this statement is not correct you must amend it as needed.

Please include this amended Role of Funder statement in your cover letter; we will change the online submission form on your behalf.

**Answer: The funder had no role in study design, data collection and analysis, decision to publish, or preparation of the manuscript.**

5. Thank you for stating the following in the Acknowledgments Section of your manuscript:

This work was supported by JSPS KAKENHI Grant Number JP 22K08976.

We note that you have provided funding information that is not currently declared in your Funding Statement. However, funding information should not appear in the Acknowledgments section or other areas of your manuscript. We will only publish funding information present in the Funding Statement section of the online submission form.

Please remove any funding-related text from the manuscript and let us know how you would like to update your Funding Statement. Currently, your Funding Statement reads as follows:

This work was supported by JSPS KAKENHI [Grant Number: JP 22K08976].

Please include your amended statements within your cover letter; we will change the online submission form on your behalf.

**Answer: As requested, we have removed the Acknowledgments section from the manuscript and included the relevant funding information in the cover letter.**

6. If the reviewer comments include a recommendation to cite specific previously published works, please review and evaluate these publications to determine whether they are relevant and should be cited. There is no requirement to cite these works unless the editor has indicated otherwise.

**Answer: We confirm that all the requested changes have been incorporated into the revised manuscript text. There were no suggested citations for previously published works.**

Reviewers' comments:

Reviewer's Responses to Questions

Comments to the Author

1. Is the manuscript technically sound, and do the data support the conclusions?

The manuscript must describe a technically sound piece of scientific research with data that supports the conclusions. Experiments must have been conducted rigorously, with appropriate controls, replication, and sample sizes. The conclusions must be drawn appropriately based on the data presented.

Reviewer #1: Yes

Reviewer #2: Yes

2. Has the statistical analysis been performed appropriately and rigorously?

Reviewer #1: Yes

Reviewer #2: No

**Answer: We appreciate the reviewer’s comment. In response, we have revised the manuscript to clarify the statistical methods used and have included the corresponding results in the Results section. We believe this revision improves the transparency and rigor of our analysis.**

3. Have the authors made all data underlying the findings in their manuscript fully available?

The PLOS Data policy requires authors to make all data underlying the findings described in their manuscript fully available without restriction, with rare exception (please refer to the Data Availability Statement in the manuscript PDF file). The data should be provided as part of the manuscript or its supporting information, or deposited to a public repository. For example, in addition to summary statistics, the data points behind means, medians and variance measures should be available. If there are restrictions on publicly sharing data—e.g. participant privacy or use of data from a third party—those must be specified.

Reviewer #1: No

Reviewer #2: No

**Answer: We have uploaded the complete dataset to Figshare and have made it publicly accessible in accordance with the journal’s open data policy. The dataset can be accessed via the following DOI: 10.6084/m9.figshare.30738818.**

4. Is the manuscript presented in an intelligible fashion and written in standard English?

PLOS ONE does not copyedit accepted manuscripts, so the language in submitted articles must be clear, correct, and unambiguous. Any typographical or grammatical errors should be corrected at revision, so please note any specific errors here.

Reviewer #1: Yes

Reviewer #2: No

**Answer: The revised manuscript has been edited for English language by Editage, with corrections performed by a native speaker.**

5. Review Comments to the Author

Please use the space provided to explain your answers to the questions above. You may also include additional comments for the author, including concerns about dual publication, research ethics, or publication ethics. (Please upload your review as an attachment if it exceeds 20,000 characters)

Reviewer #1: I want to start by acknowledging that you have tackled a very practical question in segmentectomy. The overall experimental setup is clinically relevant. Your focus on quantitative ICG behavior is refreshing and useful. I’m sharing the comments below in a constructive spirit:

1. You already present the 5–10 mg/L “sweet spot” and the adequacy of 300 mL/h quite clearly. It may help readers if you end the Abstract and the Discussion with one practical recommendation (something like a clear “this is what we would do clinically based on our findings”).

**Answer: We appreciate your valuable suggestion. In response, we have added a clear practical recommendation to the end of both the Abstract and the Discussion sections. Specifically, we now state that maintaining blood ICG concentration within 5–10 mg/L for strong fluorescence is best achieved with a relatively slow infusion rate of 300 mL/h, which we recommend as a practical approach in clinical practice.**

2. You explain the stepwise design, but it would help to spell out just a bit more transparently how Stage 1 influenced Stage 2. Was patient accrual sequential? And what exactly triggered the choice of conditions in Stage 2?

**Answer: Thank you for your helpful comment. In response, we have revised the Abstract to more clearly describe how the two-stage study design influenced subsequent steps. Specifically, we now state that the higher blood concentration of ICG observed in Group H during Stage 1 led us to set the dose in Stage 2 (Group F) at 0.2 mg/kg. This addition clarifies the sequential nature of patient accrual and the rationale for the conditions chosen in Stage 2.**

3. For Stage 1 you say randomization was used, but there’s no detail. Could you clarify how randomization/allocation was actually implemented? Also, for Stage 2/Group F, was every subsequent patient assigned to that group?

**Answer: The term ‘randomization’ was used to indicate that no deliberate allocation was performed; instead, cases were assigned alternately. The description has been revised to state that cases were allocated alternately. This is a complete mistranslation.**

4. The in vitro and in vivo parts are both strong, but I think one short sentence connecting concentrations for each group (L/H/F) to the 5–10 mg/L window would make your overall message feel more cohesive.

**Answer: We have added the following sentence in the last line of our “In vitro fluorescence analysis” section: "Based on these results, the target blood ICG concentration in the clinical trial was set at 5–10 mg/L."**

5. The marking success rate didn’t differ significantly between groups, so the Discussion statement about ICG concentration “significantly affecting” area identification probably needs softening.

**Answer: Thank you for your suggestion. We have revised the discussion sentence as follows: The intersegmental plane identification rate in daily clinical practice may be expected to improve by adopting a method of administration that stabilizes the blood concentration and avoids unnecessarily high concentrations.**

6. Several findings hover near significance (e.g., P=0.07) but are described a bit too confidently. I’d suggest framing them more as exploratory/suggestive trends.

**Answer: Thank you for your suggestion. The expression was revised by presenting the two observation items separately, which are described below: In the low-dose group, ICG blood concentration tended to decrease, and fluorescence intensity appeared to decline over time.**

7. Since you rely heavily on the DDG 3000 system, consider providing a brief note about its validation/calibration (with a reference if available).

**Answer: We appreciate the reviewer’s valuable comment. In response, we have added a description of the principle of pulse dye densitometry in the Methods section and provided appropriate references to support this explanation.**

8. Some mechanistic explanations (mixing, bleeding risk, central vs peripheral injection) read a little stronger than what your data directly evaluate. You may just soften the tone or label them as hypotheses.

**Answer: We appreciate the reviewer’s comment. As suggested, we recognized that this discussion might be misleading. Therefore, we have removed the corresponding section from the manuscript to avoid potential misunderstanding.**

9. You mention doing a post-hoc power analysis but don’t report the results. Consider providing a quick sentence summarizing it. Also, please make clear where Bonferroni adjustments are applied.

**Answer: In the Statistical Analysis section, we clarified that the Bonferroni correction was applied for pairwise comparisons when a significant difference was observed among the three groups. In addition, we explained the settings used for the post-hoc power analysis in the Methods, and summarized the results of this analysis in the Results section.**

Reviewer #2: Review of the paper "Evaluation of the effects of indocyanine green administration dose and speed on blood concentration and fluorescence in thoracoscopic segmentectomy". The authors presented a study with two phases: the first in vitro and the second in vivo evaluating the best dose and speed of ICG for identifying the intersegmental plane during segmentectomy. The authors found that the dose of 0.2 mg/kg administered at high speed is associated with a higher concentration of blood ICG and a better visualization of the intersegmental plane. In my opinion, the study is good, but with limited interest because the ICG dose is quite standardized, effective at high dose and without complications or patients risk because the safety profile of the drug is high.

I suggest clarifying some aspects:

1) how was the number of patient selected?

2) I suggest including the type of segmentectomies perfomed

3) I suggest using the interquartile range associated with the median

**Answer: As the reviewer correctly pointed out, although higher doses and faster administration speeds result in higher blood concentrations, these do not necessarily translate into clearer fluorescence or intersegmental visualization. In fact, rapid administration may even have adverse effects. This is the main point of our study, and we apologize for any lack of clarity in the initial manuscript. To address this, we have added the recommended dose and administration speed to both the Abstract and Discussion sections.**

1) **This study was conducted as a pilot study, and we intended to calculate the statistical power based on these results. As the reviewer correctly pointed out, the description was unclear. Therefore, we have added a post-hoc power analysis to clarify the limitations of the statistical power.**
2) **In our institution, the target segments are diverse, and listing each case individually would result in an overly complex figure. Therefore, we have provided a data sheet for reference (DOI: 10.6084/m9.figshare.30738818.). Furthermore, as previous reports have indicated that the fluorescence appearance of ICG differs between upper and lower segments, we did not present them separately. However, statistical confirmation showed that all groups included the same proportion, with six cases in the upper lobe and four cases in the lower lobe.**
3) **All values are presented as the median (interquartile range).**

6. PLOS authors have the option to publish the peer review history of their article (what does this mean?). If published, this will include your full peer review and any attached files.

If you choose “no”, your identity will remain anonymous but your review may still be made public.

Do you want your identity to be public for this peer review? For information about this choice, including consent withdrawal, please see our Privacy Policy.

Reviewer #1: Yes: Savvas Lampridis

Reviewer #2: Yes: Stefano Bongiolatti

[NOTE: If reviewer comments were submitted as an attachment file, they will be attached to this email and accessible via the submission site. Please log into your account, locate the manuscript record, and check for the action link "View Attachments". If this link does not appear, there are no attachment files.]

To ensure your figures meet our technical requirements, please review our figure guidelines: https://journals.plos.org/plosone/s/figures

You may also use PLOS’s free figure tool, NAAS, to help you prepare publication quality figures: https://journals.plos.org/plosone/s/figures#loc-tools-for-figure-preparation.

NAAS will assess whether your figures meet our technical requirements by comparing each figure against our figure specifications.

In compliance with data protection regulations, you may request that we remove your personal registration details at any time. (Remove my information/details). Please contact the publication office if you have any questions.
