# Supplementary material for: Evaluation of the effects of indocyanine green administration dose and speed on blood concentration and fluorescence in thoracoscopic segmentectomy
Source: PLoS One. 2026 Mar 26;21(3):e0345951. doi: 10.1371/journal.pone.0345951 (PMC13020837; doi:10.1371/journal.pone.0345951)
Supplement: Attachment — Submitted filename: Response to Reviewers.docx [file pone.0345951.s002.docx]

**Responses to Reviewers**

**Reviewer #1**

1. You have correctly clarified that patients were allocated alternately rather than randomly, and I appreciate the correction of the earlier wording. However, I believe this point still needs to be handled more clearly in the manuscript itself. Please describe this as non-random allocation and add a brief acknowledgment of the potential for temporal or secular-trend bias, given the sequential nature of Stage 2 following Stage 1.

Answer

　We appreciate the reviewer’s helpful suggestion. In accordance with your comment, we have revised the Methods section to explicitly state that patients were non-randomly allocated in an alternating manner within each stage. We also added a brief explanation acknowledging the potential for temporal or secular‑trend bias due to the sequential implementation of Stage 1 followed by Stage 2.

　Specifically, we added the following sentence to the Methods section:

　“Patients were non-randomly allocated in an alternating manner within each stage. Because Stage 1 and Stage 2 were conducted sequentially, we also recognized the potential for temporal or secular‑trend bias arising from changes over time in patient characteristics or clinical practice. However, the primary purpose of this design was exploratory dose and rate comparison.”

　In addition, we incorporated the following statement into the Limitations section of the 　　Discussion to further clarify the potential impact on interpretation:

　“This study used a non-random allocation method and sequential enrollment across Stage 1 and Stage 2. Therefore, the possibility of temporal or secular‑trend bias cannot be excluded, as changes over time in patient background, perioperative management, or clinical practice may have influenced the observed outcomes.”

　We believe these revisions address the reviewer’s concern and improve the clarity and transparency of the study design.

2. Although you now state that Bonferroni correction was applied for pairwise comparisons, it still seems unclear to me whether the reported “significant” p-values are adjusted or unadjusted. For example, p-values around 0.03 would not remain significant after Bonferroni correction for three pairwise tests. Please clarify this. Either report Bonferroni-adjusted p-values for pairwise comparisons or distinguish between overall test significance and exploratory/unadjusted pairwise findings.

Answer

　Thank you for pointing this out. We apologize for the lack of clarity in the previous version. All p‑values reported for the pairwise comparisons in the three‑group analysis are Bonferroni-adjusted values calculated directly by the statistical software. Therefore, the p‑values around 0.03 shown in the Results section represent adjusted p‑values and remain statistically significant after correction. To avoid misunderstanding, we have revised the Methods and Results sections to explicitly state that Bonferroni‑adjusted p‑values are reported for all pairwise comparisons. In response to your suggestion, we re‑examined all statistical procedures, including the pairwise comparisons and the evaluation of blood concentration and fluorescence intensity. We confirmed that the statistical software outputs Bonferroni‑adjusted p‑values for the pairwise tests, and we have corrected the manuscript to clearly indicate that all reported p‑values are adjusted values.

3. I think there is an inconsistency between the rebuttal and the manuscript regarding how data are summarised. In some places you state that values are presented as median (IQR), while elsewhere the Methods describe medians with min–max. Please standardise this.

Answer

　Thank you for pointing out this inconsistency. In response to your comment, we carefully reviewed the manuscript and standardised all summary statistics to be presented as median (IQR). We revised both the Methods section and all relevant tables and text to ensure consistency throughout the manuscript.

　We appreciate your attention to detail, which has helped improve the clarity and uniformity of our data presentation.

4. Your response explaining the distribution of upper versus lower segmentectomies is reasonable, but I think this information should be summarised in the manuscript itself rather than in the response letter or external dataset only (even one sentence in the baseline characteristics or limitations).

Answer

　Thank you for this helpful suggestion. In response to your comment, we have incorporated this information directly into the manuscript. Specifically, we added the distribution of upper‑ versus lower‑lobe segmentectomies to the baseline characteristics table to ensure that readers can easily confirm the balance between groups.

　In addition, we added a brief statement to the Limitations section noting that although the resected segments varied, the corresponding lung lobes were evenly distributed (six upper‑lobe and four lower‑lobe cases), thereby reducing the likelihood of lobe‑level anatomical bias.

　We believe these additions improve the transparency and completeness of the manuscript.
